# Right Ventricular Dysfunction in Cardiac Anesthesia: Perioperative Assessment and Underlying Mechanisms

**DOI:** 10.31083/RCM26286

**Published:** 2025-02-12

**Authors:** Kotaro Hori, Ryota Watanabe, Shogo Tsujikawa, Hideki Hino, Tadashi Matsuura, Takashi Mori

**Affiliations:** ^1^Department of Anesthesiology, Osaka Metropolitan University Graduate School of Medicine, 545-8586 Osaka, Japan

**Keywords:** right ventricular function, cardiac anesthesia, echocardiography, pulmonary artery catheters

## Abstract

The importance of right ventricular (RV) function has often been overlooked until recently; however, RV function is now recognized as a significant prognostic predictor in medically managing cardiovascular diseases and cardiac anesthesia. During cardiac surgery, the RV is often exposed to stressful conditions that could promote perioperative RV dysfunction, such as insufficient cardioplegia, volume overload, pressure overload, or pericardiotomy. Recent studies have shown that RV dysfunction during cardiac anesthesia could cause difficulty in weaning from cardiopulmonary bypass or even poor postoperative outcomes. Severe perioperative RV failure may be rare, with an incidence rate ranging from 0.1% to 3% in the surgical population; however, in patients who are hemodynamically unstable after cardiac surgery, almost half reportedly present with RV dysfunction. Notably, details of RV function, particularly during cardiac anesthesia, remain largely unclear since long-standing research has focused predominantly on the left ventricle (LV). Thus, this review aims to provide an overview of the current perspective on the perioperative assessment of RV dysfunction and its underlying mechanisms in adult cardiac surgery. This review provides an overview of the basic RV anatomy, physiology, and pathophysiology, facilitating an understanding of perioperative RV dysfunction; the most challenging aspect of studying perioperative RV is assessing its function accurately using the limited modalities available in cardiac surgery. We then summarize the currently available methods for evaluating perioperative RV function, focusing on echocardiography, which presently represents the most practical tool in perioperative management. Finally, we explain several perioperative factors affecting RV function and discuss the possible mechanisms underlying RV failure in cardiac surgery.

## 1. Introduction

Importantly, the significance of the right ventricular (RV) function in medically managing cardiovascular diseases and cardiac anesthesia is now being recognized since, until recently, the RV function received much less attention than the left ventricular (LV) function [1, 2, 3, 4]. This overlooking of the RV may have originated from the Fontan procedure, whereby the circulatory system is established without a functional RV [5]. Yet, for whatever reason, cardiology research mainly focused on the LV, with the RV even noted as the “forgotten chamber” [6]. However, the important role of RV function has recently been shown in cardiovascular physiology [7, 8], and numerous studies have suggested the prognostic impact of the RV in cardiovascular diseases, even when LV global function is preserved [9, 10]. During cardiac surgery, the RV is often exposed to stressful conditions that could lead to perioperative RV dysfunction, such as insufficient cardioplegia, volume overload, pressure overload, or pericardiotomy. Recent studies have further shown that RV dysfunction during cardiac anesthesia could cause difficulty in weaning from cardiopulmonary bypass (CPB) or even poor postoperative outcomes [11, 12, 13, 14, 15]. Severe perioperative RV failure may be relatively rare, with an incidence rate ranging from 0.1% to 3% in the surgical population [3, 16]. However, in patients who are hemodynamically unstable after cardiac surgery, almost half reported RV dysfunction [17]. Moreover, in patients with advanced heart failure (HF) who underwent implantation of an LV assist device (LVAD), RV failure occurred in approximately 20–30% of patients [18].

Owing to the previous long-term focus on LV function, research on RV function remains relatively new, with limited clinical evidence regarding perioperative RV function. Therefore, this review aims to understand the current perspective on perioperative RV function in adult cardiac surgery to facilitate future research in this field. The review initially provides an overview of the basic RV anatomy, physiology, and pathophysiology, which will aid understanding of the perioperative RV function. The most challenging aspect of studying the RV in a perioperative setting is accurately assessing RV function using the limited modalities available in cardiac surgery. We then summarize the current methods for evaluating perioperative RV function, focusing on echocardiography, the most practical tool in cardiac anesthesia. Finally, we explain several perioperative factors affecting RV function and discuss the possible mechanisms underlying perioperative RV failure.

## 2. RV Anatomy, Physiology and Pathophysiology

The anatomy of the RV is more complicated than that of the LV, which makes accurately assessing its function more difficult; this is one of the main reasons why the RV function remains poorly understood. While the shape of the LV has been described as a rugby ball, the RV is more uniquely shaped, with the shape roughly described as triangular, although the shape could appear crescent-shaped when viewed in cross-section [19]. The volume of the RV is 10–15% greater than that of the LV; however, because the free wall in the RV is thinner, the weight of the RV is approximately 1/6 to 1/3 less than the LV [20, 21]. Thus, because of the larger (diastolic) volume of the RV, the ejection fraction (ejection volume/diastolic volume) would be lower in right ventricular ejection fraction (RVEF) than left ventricular ejection fraction (LVEF) [3]. In contrast, the compliance of the RV is higher than that of the LV because of its thinner wall, making the RV relatively more tolerant to volume overload than to pressure overload [22].

The outlet of the LV forms an acute angle with its inlet; thus, the LV contracts with twisting, causing a vortex in blood outflow to eject at a sharp angle [23]. In contrast, the RV outlet forms a more obtuse angle; therefore, the blood outflow is more streamlined, and RV contraction involves a peristalsis-like motion [24, 25]. Simply, the RV has three wall motions: (1) inward movement of the free wall, (2) shortening of the long axis, and (3) traction of the free wall due to LV contraction [19]. Long-axis shortening is the most important of these three motions in healthy adults, accounting for approximately 75% of the RV contractions [26, 27]. This is largely due to the unique myocardial layers in the RV. While the LV myocardium consists of three distinct layers, the RV has two layers, circumferential and longitudinal; the longitudinal layer accounts for approximately 75% of the RV myocardium thickness [26, 27]. For this reason, many echocardiographic parameters assess longitudinal RV contraction [28, 29]. However, in pathological conditions, such as in patients with pulmonary hypertension (PH), global RV function correlates more with transverse movement than longitudinal contraction [26, 27, 30]. Similar alterations in RV contraction reportedly occur after CPB during cardiac surgery, as described in detail in Section 3 [10, 12]. The final RV traction motion caused by LV contraction is also an important contraction pattern in the perioperative management of the RV. Since the LV and RV share the septum, the LV contractions contribute 20–40% of the RV cardiac output (CO) [8, 10]. This “ventricular interdependence” can be easily assessed and is often helpful in the hemodynamic management of the RV during cardiac anesthesia.

RV function can potentially be impaired in pathophysiological conditions, such as pressure overload, volume overload, or cardiomyopathy of the RV, with or without the patient’s symptoms [7]. In this point of view, RV “dysfunction” is defined by abnormal RV functional parameters, and “failure” is defined by hemodynamic decompensation with typical clinical signs or symptoms. The European Society of Cardiology proposed staging RV dysfunction and failure from Stage 1 to 4 in the position statement of HF with preserved LVEF (HFpEF) [9]. Stage 1 is defined as at risk for right HF (RHF) without RV dysfunction and signs/symptoms, and Stage 2 is RV dysfunction without signs/symptoms. Stage 3 is RV dysfunction with prior or current signs/symptoms, and Stage 4 is with refractory signs/symptoms requiring specialized interventions. RHF can occur acutely or chronically [10]. Acute RHF is typically caused by a sudden increase in RV afterload (e.g., pulmonary embolism, acute respiratory failure) or a decrease in RV contractility (e.g., RV ischemia, acute myocarditis). RV dilation due to decreased RV stroke volume can impair LV diastolic filling, worsening systemic hypoperfusion. This represents the aforementioned ventricular interdependence from the RV to the LV. Chronic RHF is commonly caused by gradually increased RV afterload, most frequently due to left HF (LHF). Pathologically, RV myocytes in chronic RHF show similar alterations to the remodeling in LHF [31].

## 3. Assessment of RV Function in Cardiac Surgery

Owing to the complex anatomy of the RV, as described in Section 2, assessing the RV function remains clinically challenging, particularly with the limited modalities available in the perioperative setting. Cardiac magnetic resonance imaging (MRI) is considered the gold standard measurement for accurately evaluating this complex anatomy [10]. However, MRI is not feasible during cardiac surgery, at least in the current clinical setting. From this perspective, there is no gold standard for assessing RV function during cardiac surgery; therefore, the most appropriate methods available in the clinical situation should be chosen. Due to the difficulty in determining RV function, establishing a perioperative treatment of RV failure remains limited; thus, the treatments recommended for managing RV failure during cardiac anesthesia should instead remain followed [8, 10]. This section discusses the current understanding of several methods for assessing perioperative RV function and summarizes their characteristics for appropriate use (Table 1, Ref. [8, 10, 12, 28, 29]).

**Table 1.** **Parameters for assessing perioperative RV function**.

RV parameters			Characteristics
Cardiac MRI			
	RVEF		The gold standard for RV systolic function but remains unfeasible during cardiac surgery. Lower limit: 45%.
Clinical assessment during surgery			
	Direct visual RV assessment		Possible after cardiotomy, but relatively subjective assessment without evaluation of RV inferior or lateral walls.
Echocardiography			
	Global systolic function		
		D-shaped ventricular septum	A practical qualitative method that can also be quantitively measured.
		RVFAC	Missing data for RV anterior, infundibular, or inferior walls. Lower limit: 35%.
		RIMP	Upper limit: 0.4 by pulsed-wave Doppler; 0.55 by tissue Doppler.
		3D RVEF	Accurate, but still has some technological issues. Lower limit: 44% or 45%.
	Regional systolic function		Includes TAPSE, RVIVA, or strain. May not be appropriate after CPB due to changes in RV contraction pattern.
	Diastolic function		Little is known due to the angle dependency of TEE or positive ventilation during cardiac surgery.
Pulmonary artery catheters			
	RVEF		Underestimated due to recirculation of blood in the RV. Lower limit: 40%.
	RA/PCWP ratio		Usually about 0.5, but is higher in RV dysfunction.
	RVSWI		0.136 × SVI × (mPAP - RAP). Lower limit: 0.4.
Biochemical markers			BNP or cardiac troponin are elevated in RV failure, but this is not specific to the RV.
Electrocardiography			Frequently used modality with possible diagnostic capability, but not specific.

RV, right ventricle; MRI, magnetic resonance imaging; RVEF, RV ejection fraction; RVFAC, right ventricular fractional area change; RIMP, right ventricular index of myocardial performance; 3D, three-dimensional; TAPSE, tricuspid annular plane systolic excursion; RVIVA, right ventricular isovolumic acceleration; CPB, cardiopulmonary bypass; TEE, transesophageal echocardiography; RA, right atrium; RAP, right atrial pressure; PCWP, pulmonary capillary wedge pressure; RVSWI, right ventricular stroke work index; SVI, stroke volume index; mPAP, mean pulmonary artery pressure; BNP, B-type natriuretic peptides. Adapted from Harjola *et al*. [8], Konstam *et al*. [10], Silverton *et al*. [12], Rudski *et al*. [28], and Zaidi *et al*. [29].

### 3.1 Clinical Assessment

As the gold standard for assessing RV dysfunction during cardiac surgery is lacking, the first step in recognizing RV failure in current practice is often the usual clinical assessment of perioperative hemodynamic instability, with a quick estimation of RV dysfunction. For a quick echocardiographic assessment of the RV, eyeballing RV systolic function and dilatation, D-shaped septum, tricuspid regurgitation, or inferior vena cava diameter are useful parameters [14]. As mentioned above, in hemodynamically unstable patients after cardiac surgery, RV dysfunction was present in approximately half of the patients, which was simply assessed by right ventricular fractional area change (RVFAC) ≤25% or severe RV dilation [17]. Jabagi *et al*. [32] suggested that either direct visual estimation of reduced RV contraction in the surgical field, a marked decrease in RV echocardiographic parameters, or poor hemodynamic parameters in pulmonary artery catheters could achieve clinical assessment of perioperative RV dysfunction. Visual RV assessment in the surgical field is a traditional method and is often clinically useful. However, it should be noted that the visual assessment is mostly of the RV anterior motion; thus, the assessment lacks an evaluation of the inferior or lateral wall of the RV. In addition, due to the complex anatomy of the RV, visual assessment of RV function is reportedly difficult with echocardiography [33], which is often accurate when assessing LVEF [34].

### 3.2 Echocardiographic Assessment

Transesophageal echocardiography (TEE) is often available during cardiac anesthesia, is minimally invasive, and is cost-effective; thus, TEE is one of the most practical methods for assessing perioperative cardiac function [35, 36]. However, TEE is reportedly much less accurate for assessing RV function than cardiac MRI, particularly with two-dimensional echocardiography [28, 29]. This is due to the unique shape of the RV, difficulty in visualizing the endocardial border with the developed trabeculae in the RV, or difficulty in aligning the RV away from the esophagus [12, 28, 29]. In addition, because the RV wall is thinner than the LV wall, the echocardiographic parameters of the RV function are more load-dependent; therefore, pressure- and volume-loaded conditions should always be considered during examinations [28].

**Fig. 1.**
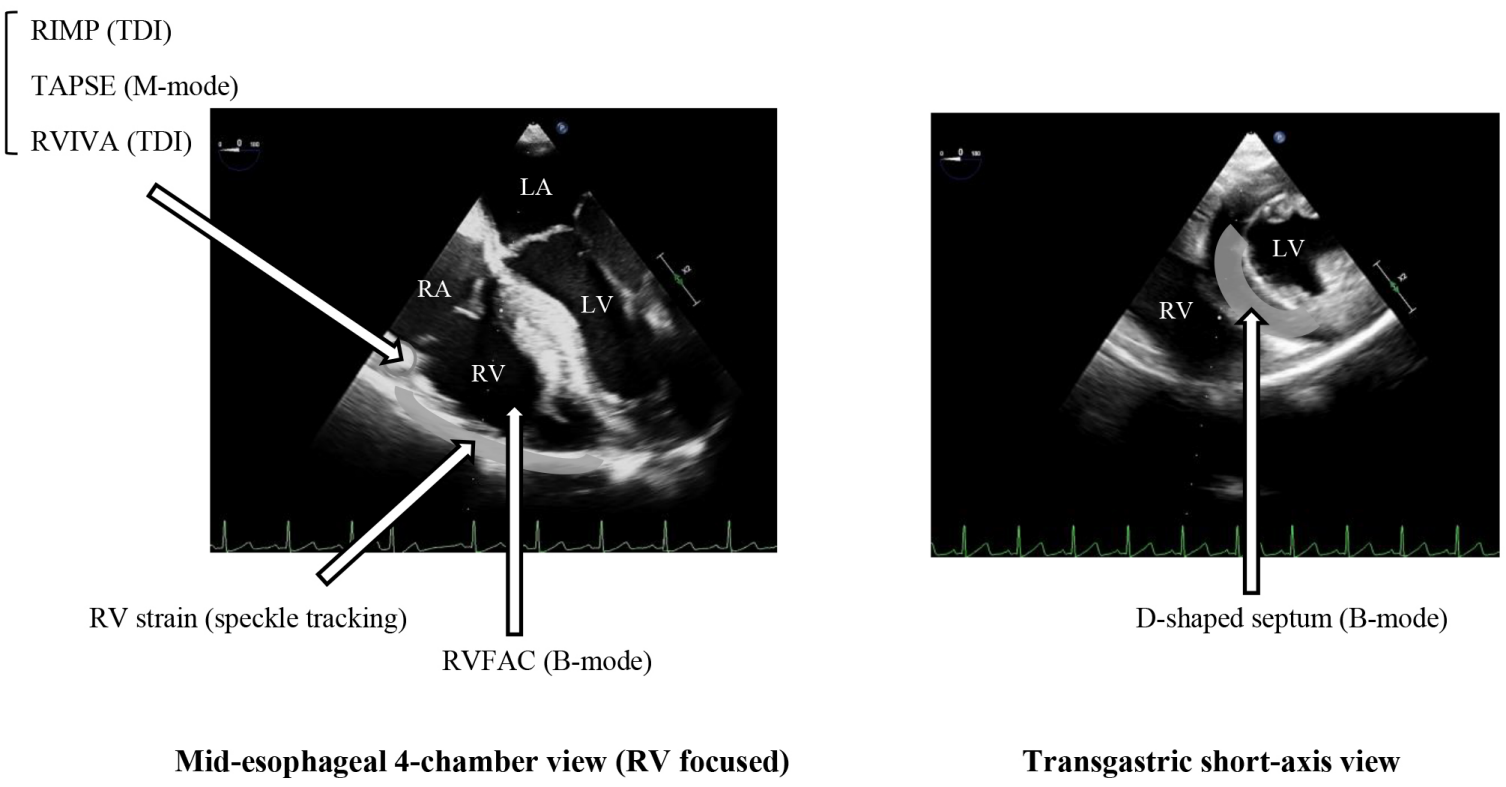
**TEE views and their locations for measuring RV echocardiographic parameters**. Many echocardiographic RV parameters can be measured in TEE at the mid-esophageal 4-chamber or transgastric short-axis view. LV, left ventricle; RV, right ventricle; RA, right atrium; TEE, transesophageal echocardiography; RIMP, right ventricular index of myocardial performance; TDI, tissue Doppler imaging; TAPSE, tricuspid annular plane systolic excursion; RVIVA, right ventricular isovolumic acceleration; RVFAC, right ventricular fractional area change; LA, left atrium.

There are two approaches for assessing RV systolic function by echocardiography: global and regional. With RVFAC as a representative example, global assessment is a conventional method for the echocardiographic assessment of systolic function. However, assessing the whole RV accurately is difficult because of its complex anatomy. Since long-axis contraction is the main component of RV systolic function in healthy adults, as mentioned above, echocardiographic regional assessment of longitudinal measures, such as tricuspid annular plane systolic excursion (TAPSE) or RV strain, is also commonly used. Fig. 1 summarizes the commonly used two-dimensional views and their measurement locations for assessing RV function in TEE.

#### 3.2.1 Global RV Systolic Function

“D”-shaped ventricular septum in the transgastric short-axis view is a commonly observed clinical sign of RV ed fidysfunction, which is a qualitative RV assessment. As mentioned above, the RV crossed section is crescent in shape; thus, the ventricular septum in the short-axis view normally has a convex shape to the RV, and the LV forms a round “O” shape. However, in an RV-overloaded condition, the ventricular septum could change to a flattened shape, creating a “D” shape with a convex shape remaining on the LV lateral wall. A flattened septum during systole suggests pressure overload in the RV, while that during diastole suggests volume overload [12]. This is a convenient qualitative method, but it can also be evaluated quantitatively by measuring the ratio of the vertical to the horizontal diameter of the LV or the ratio of the horizontal diameter of the RV to that of the LV [37, 38].

RVFAC is the method conventionally used for assessing RV systolic function and is calculated as the percentage of the end-systolic to the end-diastolic area of the RV in a mid-esophageal 4-chamber view. Although similar to the commonly used two-dimensional LVEF calculated by Simpson’s biplane method, sagittal plane measurements are unsuitable for the RV due to its unique shape [28]. Thus, the RVFAC is measured only in a single plane, which is the main limitation of this method, as data from the anterior, infundibular, or inferior walls of the RV are missing. A RVFAC <35% is considered to indicate RV dysfunction, and several studies have shown that a reduced RVFAC is associated with higher morbidity and mortality after cardiac surgery [39, 40, 41].

The right ventricular index of myocardial performance (RIMP), also known as the Tei index or myocardial performance index (MPI), is a parameter of RV systolic and diastolic function, which is calculated as the sum of the isovolumetric contraction time and isovolumetric relaxation time, divided by the ejection time. The RIMP can be obtained by pulsed-wave or tissue Doppler. Using pulsed-wave Doppler, two separate images of the tricuspid inflow and right ventricular outflow tract (RVOT) should be obtained to measure the time interval from the closure to the opening of the tricuspid valve and the ejection time through the RVOT. Tissue Doppler is generally preferred because the RIMP can be measured using a single image of the tricuspid annulus in a 4-chamber view. However, a high-quality signal is required to calculate the above parameters accurately [29]. The normal upper limit of the RIMP is reportedly 0.4 by pulsed-wave Doppler and 0.55 by tissue Doppler [28].

Three-dimensional RVEF is currently the most accurate echocardiographic method for assessing RV systolic function and correlates well with the RVEF measured by cardiac MRI with high reproducibility. As it tends to underestimate RVEF slightly compared with MRI [19, 42], the lower limit of the three-dimensional RVEF is commonly set at 44% or 45% [28, 29]; comparatively, the lower limit of RVEF for cardiac MRI is 45%. Given the complex RV anatomy, it is reasonable that three-dimensional measurement is the most accurate. However, this measurement still has some technological issues, such as potential image dropout or software availability [12, 19, 42]. However, as technological issues are likely to improve in the near future, three-dimensional RVEF may become the gold standard in the perioperative assessment of RV systolic function, given that MRI is unavailable in the operating room.

#### 3.2.2 Regional RV Systolic Function

Regional assessment of RV longitudinal contractions, such as TAPSE, RVIVA (right ventricular isovolumic acceleration), or RV strain, is clinically useful because longitudinal movement is easier to measure than assessing the global function of the RV by echocardiography, given its complex anatomy. However, the longitudinal RV measures are based on the principle that longitudinal contraction represents the major component of RV systolic function. This is true in healthy adults but may be incorrect in some pathological conditions, such as PH and cardiac surgery after CPB. Recent evidence has suggested that pericardiotomy and/or CPB during cardiac surgery often decrease RV longitudinal contraction, even when global function is preserved, with increased RV transverse shortening [43]. This alteration in RV contraction persists for approximately one week after surgery [44]. A similar reduction in longitudinal contractions is observed in patients with PH [26, 27, 30]. Although the clinical implications of changes in RV contraction remain unclear, the usefulness of regional RV assessment in cardiac surgery may be limited [10, 12].

#### 3.2.3 RV Diastolic Function

Although RV systolic function remains unclear, RV diastolic function is even less studied, particularly in cardiac surgery. This is largely due to the acoustic angle dependence of TEE or positive pressure ventilation during surgery [12]. Using transthoracic echocardiography (TTE), the trans tricuspid E/A or E/e’ ratio is the parameter most commonly used to assess RV diastolic function [28, 29]; however, it may be difficult to measure accurately during cardiac anesthesia because of the angle-dependence of TEE. For the same reason, hepatic or splenic vein Doppler imaging may be unsuitable for perioperative assessment. RV diastolic dysfunction has been reported in many types of cardiovascular diseases and is a possible independent predictor of increased mortality [45, 46, 47]. Therefore, further studies are warranted to establish reliable echocardiographic parameters for perioperative assessment of RV diastolic function.

### 3.3 Hemodynamic Assessment by Pulmonary Artery Catheters

Pulmonary artery catheters (PACs) have been widely used in cardiac surgery since the 1970s; however, many recent studies have shown that PACs do not significantly improve patient outcomes and may even be harmful because of the potential complications associated with catheter use [48, 49, 50]. Therefore, PACs are currently used much less frequently during cardiac anesthesia but remain useful tools for detailed hemodynamic monitoring. Since right arterial pressure (RAP) is easily measured using central venous catheters during cardiac anesthesia, elevated RAP is one of the most common hemodynamic signals associated with perioperative RV dysfunction. However, the elevated RAP may be derived from increased left atrial pressure, which can be evaluated by measuring pulmonary capillary wedge pressure (PCWP). When PACs are used, the right atrium (RA)/PCWP ratio may be useful for assessing RV dysfunction. This ratio is usually about 0.5 but is higher in patients with RV dysfunction [51, 52]. In patients undergoing LVAD implantation, when the diagnosis of RV failure is sufficiently clear, with presumed normal LV function with the implanted devices, a preoperative RA/PCWP ratio >0.63 predicts RV failure after surgery [53]. In addition, PCWP is useful for estimating the causes of PH. Elevated PCWP suggests post-capillary PH, not pre-capillary PH [54]. The RV stroke work index (RVSWI) is another useful parameter, which is calculated as “RVSWI = 0.136 × SVI ×(mPAP - RAP)” (SVI; stroke volume index, mPAP; mean pulmonary artery pressure). An RVSWI <4 suggests RV dysfunction and is reportedly associated with increased mortality [55, 56].

PACs provide many hemodynamic parameters, among which continuous CO, measured using the thermodilution technique, is one of the most useful parameters for perioperative hemodynamic management [54]. PACs can assess RV function specifically by estimating right ventricular end-diastolic volume (RVEDV) and RVEF using a rapid-response thermistor. In general, the thermodilution-derived RVEF underestimates the MRI-measured RVEF; thus, the lower limit of the RVEF by PACs is often set at 40%. RV physiology may explain this underestimation. An animal study revealed that the reduced temperature in the RA caused by cold fluid injection did not return to baseline within a single heartbeat [57]. In addition, MRI of the human heart demonstrated a recirculation of blood in the RV due to the phasic RV contraction pattern [25]. Although measuring the RVEF using PACs may be less accurate than MRI, it could be useful for trend monitoring, particularly in perioperative settings without reliable RV monitoring parameters.

### 3.4 Other Assessment Methods

Biochemical markers are frequently used in patients undergoing cardiac surgery, which are useful for the clinical management of HF but are not specific for RV failure [58, 59]. BNP (B-type natriuretic peptides) and cardiac troponin levels are reportedly elevated in patients with RV failure caused by acute pulmonary embolism (PE) and are associated with poor clinical outcomes [60, 61, 62]. However, both markers are also elevated in LV failure and are not sufficiently sensitive to assess the degree of dysfunction in each ventricle. Some studies have reported the development of specific biomarkers for RV dysfunction that may help evaluate and treat RV failure in the near future [10].

Electrocardiography (ECG) is often used for preoperative evaluation in cardiac surgery patients and may have diagnostic capabilities for RV dysfunction. On a 12-lead ECG, a qR pattern in V1 may indicate acute RV failure, and SI, QIII, TIII (deep S wave in I lead, and Q wave and negative T wave in III lead) is a famous pattern for pulmonary embolism [63, 64]. In addition, several studies have shown that QRS duration, particularly on the right-sided chest leads (V1, V2), correlates well with RV function and volume as evaluated using MRI [65]. Despite the potential utility of right-sided chest leads, because the chest leads are often unavailable during cardiac surgery, we recently investigated the usefulness of QRS duration on intracardiac right ventricular ECG obtained through a pacing catheter. Intravenous pacing is a useful tool in a small surgical site of minimally invasive cardiac surgery. Additionally, QRS duration in the RV has been shown to be useful for assessing RV function during cardiac surgery [66]. However, we need to be careful that ECG findings are often nonspecific, and even the famous “SI, QIII, T III” pattern is known to be seen in only about 20% of patients with pulmonary embolism [67]. Furthermore, the ECG waveform can be altered by many types of perioperative drugs and surgical stress [68, 69, 70].

## 4. RV Function in Cardiac Surgery

Although severe perioperative RV failure may be relatively rare, the incidence of mild-to-moderate RV failure remains unclear, which could have significant clinical implications. Many factors in cardiac surgery can cause perioperative RV failures, such as pre-existing RV dysfunction, pericardiotomy, CPB, mechanical ventilation, or RV volume and pressure overload. This section explains several factors affecting RV function in cardiac surgery and discusses the possible mechanisms underlying perioperative RV dysfunction.

Preoperative RV dysfunction is a possible cause of postoperative RV failure. Due to ventricular interdependence, patients with HF and reduced LVEF (HFrEF) often have RV dysfunction. In a meta-analysis, the prevalence of RV systolic dysfunction in HFrEF was as high as 48% [71]. Similar to patients with HFrEF, RV dysfunction is common in patients with HFpEF, with a prevalence of approximately 20%, as confirmed using MRI [72]. In patients with inferior wall myocardial infarction (MI), 30–50% are known to have MI in the RV [73, 74]. Hemodynamic compromise is less common in patients with right ventricular myocardial infarction (RVMI) than in those with LVMI but still occurs in 25–50% of patients with RVMI [75]. Valvular disease can also directly or indirectly affect RV function. In patients undergoing corrective surgery for isolated tricuspid regurgitation, the effective RVEF measured using MRI was reduced in more than half of the population [76]. In patients with left-sided valvular disease undergoing surgical treatment, preoperative RV dysfunction was observed in approximately 20% of the population [77]. Particularly, RV dysfunction was reportedly present in about 30% of mitral regurgitation cases [78]. PH, with or without valvular disease, is another well-known cause of RV failure. Since many factors in cardiac surgery could increase pulmonary vascular resistance, such as hypoxia, hypercapnia, acidosis, hypothermia, or anemia, we should carefully monitor RV function and optimize the perioperative factors to prevent perioperative RV failure [79, 80].

Intraoperative factors in cardiac surgery can directly reduce RV function, but among them, CPB appears to be the major cause of perioperative RV dysfunction. Several studies have shown that a long CPB duration strongly predicts RV failure during cardiac anesthesia [81, 82, 83]. Further, differences in cardioplegia can affect RV function after CPB. Warm cardioplegia (generally 34–35 °C) might yield better RV function than cold cardioplegia (less than 4 °C) after CPB [84, 85]. During CPB, many types of cytokines are induced, and endothelin-1, in particular, may play an important role in postoperative RV dysfunction through vasoconstriction of the pulmonary arterioles [86]. Coronary air embolism and acute graft occlusion are also well-known causes of RV failure during cardiac surgery. In addition to CPB, pericardiotomy itself, for example, could cause non-physiological patterns of RV filling, leading to possible RV dysfunction [87, 88, 89]. RV volume and pressure overload during cardiac surgery also potentially cause postoperative RV dysfunction [90, 91]. General anesthetics also appear to affect RV function negatively [92, 93, 94]. Although it is difficult to accurately evaluate the effects of anesthetics during general anesthesia because the above-mentioned intraoperative factors could also affect RV function, inhalational anesthetics, including sevoflurane and isoflurane, or propofol, have reportedly reduced echocardiographic RV parameters. Some studies compared the effects of inhalational and intravenous anesthetics on RV parameters; however, these results were inconsistent [95, 96, 97]. Although the specific mechanisms underlying postoperative RV dysfunction remain unclear, the above-mentioned perioperative factors might collectively affect RV function and cause RV failure during cardiac anesthesia.

## 5. Conclusions

Although much information on RV function, particularly during cardiac anesthesia, requires to be elucidated, this field is clearly developing, as summarized in this review. However, the major issue in this area is that the perioperative assessment of RV function has yet to be established without a gold standard that can replace cardiac MRI. Even if cardiac MRI were available in the operating room, it may not be useful for evaluating RV function during cardiac anesthesia. Currently, there are many parameters to assess perioperative RV function, yet we should understand their characteristics and choose the most suitable parameters in the perioperative setting to ensure their proper use. Hence, further technological progress and new ideas are needed to assess perioperative RV function accurately and practically. Real-time 3D RVEF or RV-specific biomarkers may be the most feasible methods. Once a gold standard for perioperative RV assessment has been established, more attention should be paid to the perioperative treatment of RV failure, which is being investigated in medical management but remains in its infancy. Elucidating the mechanisms through which perioperative RV dysfunction occurs may also help to improve its treatment and prevention. This review summarizes the current status and problems associated with perioperative RV function in cardiac anesthesia. Among these various issues, improving perioperative RV assessment is the most important, and we may be able to contribute to a better understanding and treatment of perioperative RV failure. Following an extended research period on LV function in cardiology and cardiac surgery, research should instead focus on the RV function.
